# Dr. Joseph Tottenham Cook

**Published:** 1915-02

**Authors:** 


**Dr. Joseph Tottenham Cook,** Cleveland Homoeopathic Medical College, 1881, died at his home in Buffalo, January 18, 1915, aged 59. The cause of death was facial erysipelas. Dr. Cook was born in Ludlowville, N. Y., the son of Rev. Philos G. Cook but as his family moved to Buffalo during his early childhood, both his father and himself were identified with the life of Buffalo, in their respective professions and by their strong personal influence. After graduating in medicine, Dr. Cook pursued his medical studies in London and at the Royal Imperial General Hospital of Vienna, and began practice in Buffalo in 1882. He was a member of the American Institute of Homoeopathy, of the State and County Homoeopathic Medical Societies and, at the time of his death, was Vice-President of the Buffalo Homoeopathic Hospital and the Clinical Club. His extra-professional interests are indicated by his membership in the following organizations: Buffalo Historical Society, N. Y. State Historical Association, Sons of St. George, Sons of the Revolution, Society of Colonial Wars, Sons of Veterans—his father being commonly known as "Chaplain" Cook, in affectionate and persistent remembrance of his labors during the Civil Wark.

Dr. Cook was a highly successful and highly skilled practitioner of medicine, loyal to his school of medicine but in so broad a spirit as to warrant not only the respect but the fraternal regard, personally and professionally, of the entire medical profession. He was an upright man, a good citizen, a scholarly gentleman.

